# Staged non-bridging circular external fixation with sequential osteoporosis therapy for MRSA-infected distal radius non-union: a case report

**DOI:** 10.1186/s12891-026-09865-z

**Published:** 2026-04-25

**Authors:** Koji Nozaka, Tsuyoshi Shirahata, Yusuke Yuasa, Shuntaro Harata, Ryota Kimura, Manabu Akagawa, Naohisa Miyakoshi

**Affiliations:** Department of Orthopaedic Surgery, Akita University Graduate School of Medicine, 1-1-1 Hondo, Akita, 010-8543 Japan

**Keywords:** Distal radius fracture, Infected non-union, Circular external fixation, Non-bridging fixation, MRSA infection, Severe osteoporosis, Teriparatide, Zoledronic acid, Limb salvage, Case report

## Abstract

**Background:**

Infected distal radius non-union in elderly patients with severe osteoporosis is particularly challenging because infection eradication, stable fixation, and preservation of wrist function must be achieved simultaneously. Conventional internal fixation in an infected osteoporotic field carries a high risk of recurrent infection and fixation failure, whereas bridging external fixation may result in prolonged immobilization and functional deterioration. We report a case of methicillin-resistant Staphylococcus aureus (MRSA)-infected distal radius non-union treated with staged non-bridging circular external fixation combined with sequential osteoporosis therapy.

**Case presentation:**

An 85-year-old woman with severe osteoporosis developed MRSA-infected distal radius non-union after failed plate fixation. A staged treatment strategy was employed. In the first stage, non-bridging circular external fixation was applied, followed by implant removal, radical debridement, and vancomycin-loaded hydroxyapatite implantation, together with systemic antibiotic therapy. Immediate wrist mobilization and low-intensity pulsed ultrasound therapy were initiated. After infection control, second-stage autologous cancellous bone grafting was performed. Weekly teriparatide therapy had been initiated preoperatively and was continued for 18 months, followed by annual intravenous administration of zoledronic acid. Radiographic union was achieved 3 months after bone grafting. At final follow-up (5 years), the Disabilities of the Arm, Shoulder, and Hand (DASH) score was 10.7, with preserved wrist motion and full functional independence. No recurrence of infection or refracture was observed.

**Conclusions:**

Staged non-bridging circular external fixation combined with sequential osteoporosis therapy may represent a potential salvage approach for infected distal radius non-union in severely osteoporotic elderly patients. This approach enables infection control, stable fixation, and early mobilization while preserving wrist function. However, as this is a single case treated with multiple simultaneous interventions, the findings should be interpreted cautiously and considered hypothesis-generating.

## Background

Distal radius fractures are among the most common fragility fractures in elderly patients. Although volar locking plate fixation is widely used, postoperative infection in the setting of severe osteoporosis remains a serious complication because both infection eradication and stable fixation must be achieved in biologically and mechanically compromised bone. In such cases, repeated internal fixation may be unreliable because hardware reimplantation into an infected osteoporotic metaphysis increases the risks of recurrent infection and fixation failure. Conversely, bridging external fixation may provide temporary stability but often requires prolonged wrist immobilization, which can compromise functional recovery in elderly patients [1, 2].

Circular non-bridging external fixation using tensioned transosseous wires has been reported to provide stable fixation while preserving wrist motion in unstable distal radius fractures, and anatomical studies have supported the feasibility of safe wire insertion around the distal radius [1, 2]. However, reports specifically addressing infected distal radius non-union in patients with severe osteoporosis remain very limited.

In addition to local mechanical and infection-related factors, systemic skeletal fragility is an important component of treatment planning in elderly patients. Sequential anabolic-to-antiresorptive therapy is biologically attractive because it aims first to stimulate bone formation and then to preserve gained bone mass [3–5]. Low-intensity pulsed ultrasound has also been used as an adjunct for fracture healing, although the specific contribution of each adjunct cannot be determined in an individual case treated with multiple simultaneous interventions [6–8].

Here, we report a case of MRSA-infected distal radius non-union in severe osteoporosis treated with staged non-bridging circular external fixation, staged debridement and bone grafting, and sequential osteoporosis therapy. The novelty of this report lies not in any single component alone, but in the practical integration of infection control, wrist-preserving fixation, and systemic bone health optimization in one highly challenging clinical setting.

## Case presentation

**Fig. 1:**
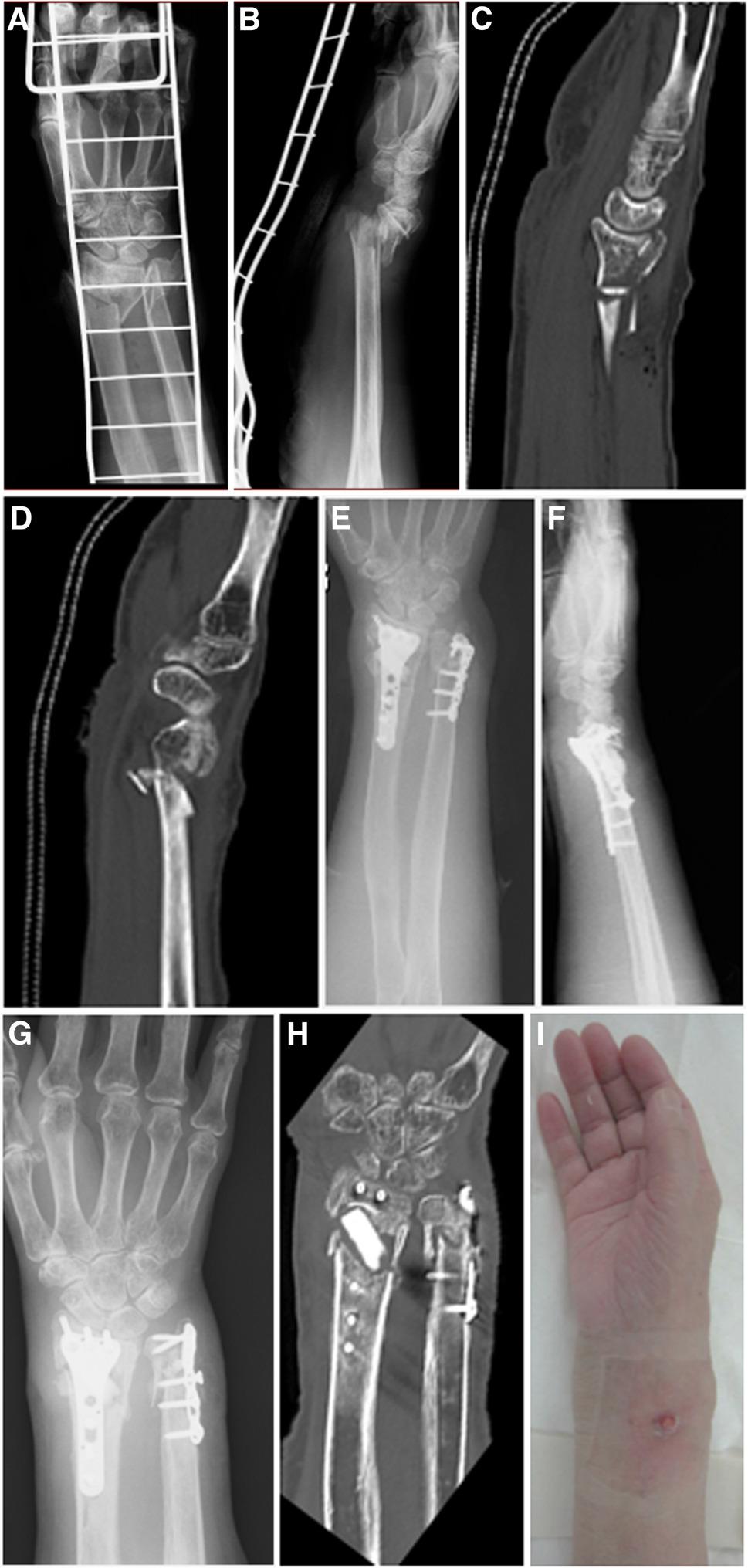
Clinical course from injury to referral. **A** Anteroposterior radiograph at the time of injury demonstrating an AO/OTA 23-C3 distal radius fracture. **B** Lateral radiograph at the time of injury showing metaphyseal comminution and displacement. **C** Immediate postoperative anteroposterior radiograph after volar plate fixation at the previous hospital. **D** Immediate postoperative lateral radiograph. **E** Three-month postoperative anteroposterior radiograph demonstrating persistent non-union. **F** Coronal CT image at three months showing incomplete osseous bridging and metaphyseal bone loss. **G** Clinical photograph at referral showing a draining sinus on the volar aspect of the wrist arising from the distal radial non-union site

An 85-year-old woman sustained an AO/OTA 23-C3 open distal radius and ulna fracture (Gustilo type I) after trauma (Fig. 1A, B). Initial treatment at another hospital consisted of plate fixation (Fig. 1C, D). However, postoperative recovery was complicated by persistent infection and non-union. Three months after the index procedure, radiography and computed tomography demonstrated persistent non-union with bone loss (Fig. 1E, F, H). She was referred to our institution 6 months after the original surgery because of a draining sinus over the volar wrist (Fig. 1I), raising concern for infected non-union after failed internal fixation. Bone mineral density assessment demonstrated severe osteoporosis, with YAM 49% and a femoral neck T-score of -3.4. Weekly teriparatide therapy had been initiated preoperatively.

The treatment goals were as follows: (1) eradication of infection, (2) stable fixation of the distal osteoporotic fragments, (3) preservation of wrist and forearm function through early mobilization, and (4) optimization of the underlying osteoporotic bone environment.

### First-stage surgery

Because revision internal fixation in an infected field with severe metaphyseal osteoporosis was considered to carry a high risk of recurrent infection and fixation failure, we selected staged non-bridging circular external fixation. A hybrid non-bridging construct was applied before plate removal in order to maintain stability throughout the debridement procedure. Two crossing 1.8-mm transosseous wires were inserted into the distal fragment and tensioned to 130 kg on a 140-mm ring. One wire was introduced from the interval between the extensor carpi radialis longus and extensor pollicis brevis, directed toward the dorsal aspect of the flexor carpi ulnaris on the ulnar side. The second wire was inserted from the volar side of the abductor pollicis longus toward the palmar aspect of the extensor digiti minimi on the ulnar dorsal side. All wires were inserted using a mini-open technique with small skin incisions and direct visualization at both entry and exit points to minimize the risk of tendon and soft-tissue injury. The proximal segment was stabilized using two 4-mm half-pins connected to the circular frame via rod constructs. Olive wires were not used because the soft-tissue envelope was thin and the olive ends were considered likely to interfere with wound closure.

This configuration was selected to achieve sufficient stability of the small osteoporotic distal fragment while preserving wrist motion, whereas bridging fixation was avoided because of the risk of prolonged immobilization and functional deterioration.

After the frame had been secured, both plates were removed and extensive debridement was performed. Vancomycin-impregnated hydroxyapatite was inserted into the defect. Intraoperative cultures obtained from the non-union site yielded MRSA, confirming persistent infection. Intravenous anti-MRSA therapy was initiated at admission and consisted of daptomycin administered at a dose of 6 mg/kg once daily for 6 weeks under the supervision of an infectious disease specialist. No further intravenous antibiotic therapy was administered after completion of this course. At presentation, inflammatory markers were elevated, with a C-reactive protein level of 2.03 mg/dL and a white blood cell count of 9,800/µL. At 2 weeks after surgery, the C-reactive protein level had decreased to 0.17 mg/dL and the white blood cell count to 4,300/µL. Thereafter, both values remained within normal ranges, and no recurrence of infection was observed.

**Fig. 2:**
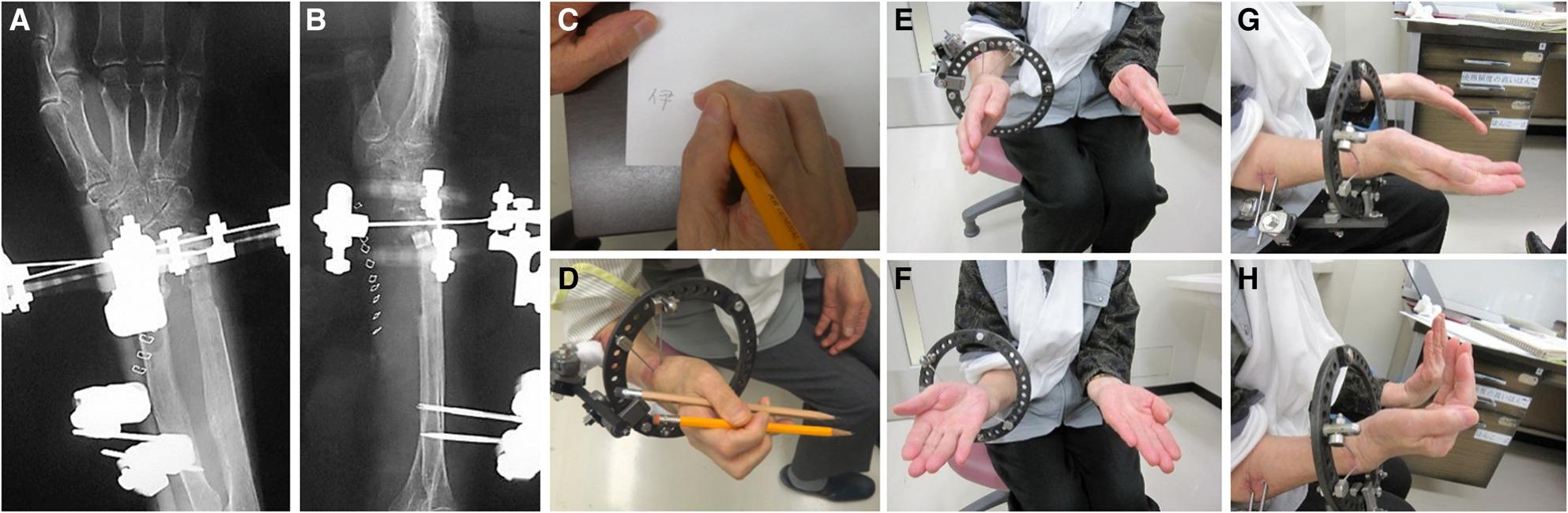
Clinical and radiographic findings after first-stage surgery. **A** Immediate postoperative anteroposterior radiograph showing non-bridging circular external fixation. **B** Immediate postoperative lateral radiograph. **C** Writing activity performed during external fixation. **D** Chopstick use during external fixation. **E** Active forearm pronation. **F** Active forearm supination. **G** Active wrist dorsiflexion. **H** Active wrist palmar flexion

Daily LIPUS therapy was initiated according to the manufacturer’s protocol for 20 min per day. Because preservation of wrist motion was one of the central aims of treatment, immediate active wrist and forearm mobilization was encouraged after surgery. The patient was also encouraged to continue functional daily activities, including writing and chopstick use, during external fixation (Fig. 2C, D), and she was able to continue ordinary home life during the treatment period. Postoperative pin-site care followed a simple protocol. After suture removal from the plate-removal incision at 2 weeks postoperatively, open showering was permitted, and the pin sites were left uncovered. Oral antibiotics were administered for 5 days only when mild pin-site infection was observed during follow-up.

### Second-stage surgery

**Fig. 3:**
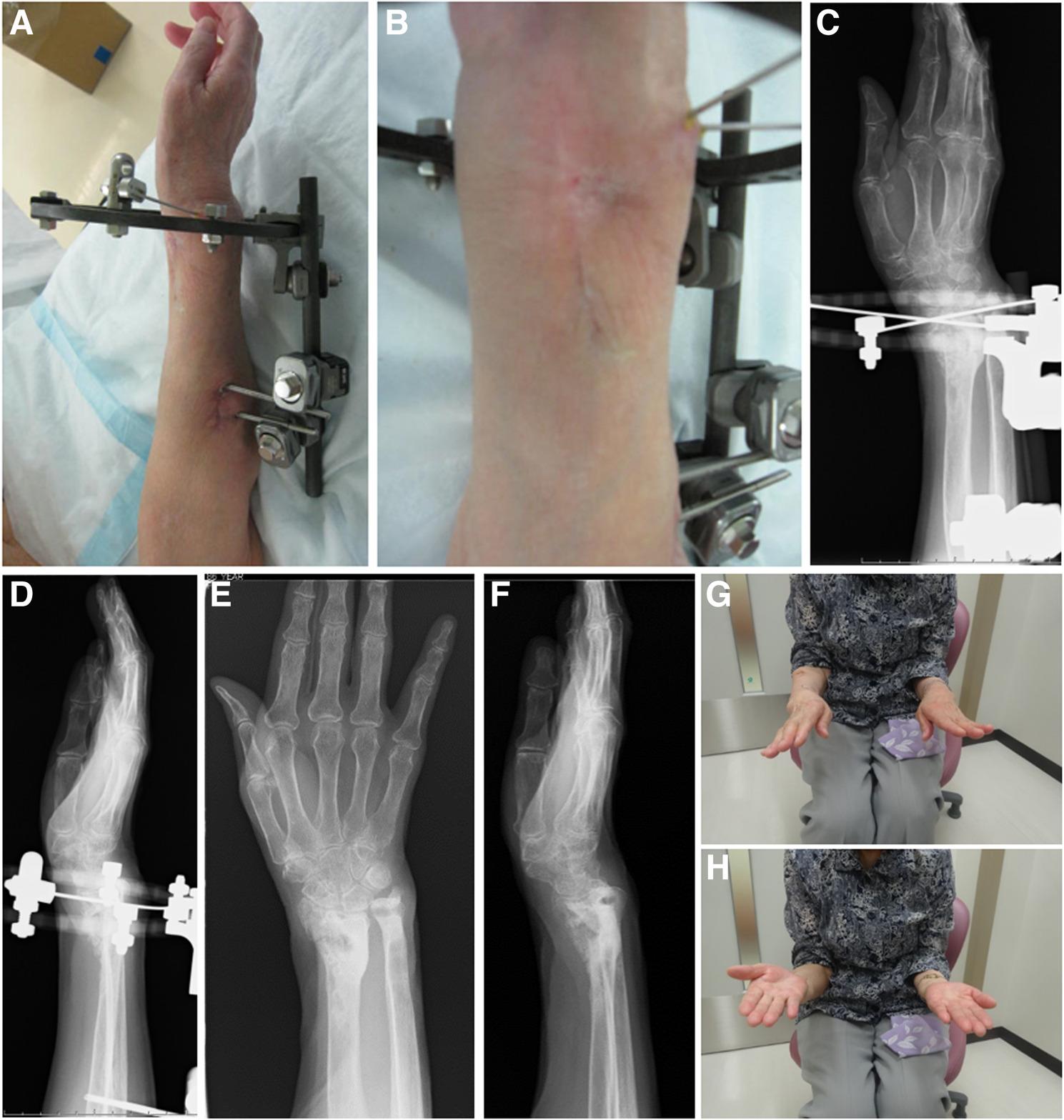
Clinical and radiographic findings after second-stage surgery and follow-up. **A**, **B** Healed sinus tract six weeks after first-stage surgery. **C** Anteroposterior radiograph after second-stage autologous cancellous bone grafting. **D** Lateral radiograph after second-stage surgery. **E** Anteroposterior radiograph demonstrating bony union prior to fixator removal. **F** Lateral radiograph confirming union. **G** Forearm pronation after fixator removal. **H** Forearm supination after fixator removal

Six weeks after the first-stage procedure, the sinus tract had healed and inflammatory markers had normalized (Fig. 3A, B). The antibiotic-loaded hydroxyapatite was removed. The non-union site was refreshed until punctate bleeding was observed, and autologous cancellous bone grafting was then performed while maintaining the circular fixator (Fig. 3C, D). Intraoperative cultures obtained at the second-stage surgery were negative, indicating microbiological resolution of the infection.

Three months after bone grafting, radiographs demonstrated bony union, and the external fixator was removed (Fig. 3E, F). At final follow-up, forearm pronation and supination had been preserved (Fig. 3G, H). The DASH score measured at 12 months after fixator removal was 10.7, consistent with excellent upper-extremity function. Objective functional outcomes at final follow-up were as follows: wrist dorsiflexion, 20 degrees on the affected side versus 30 degrees contralaterally; palmar flexion, 40 degrees versus 50 degrees; forearm pronation, 60 degrees versus 70 degrees; and supination, 80 degrees versus 90 degrees. Grip strength was 12 kg on the affected side and 15 kg contralaterally. Pain assessed using a visual analogue scale improved from 50/100 at presentation to 5/100 at final follow-up. The patient achieved full independence in activities of daily living and was able to live alone without assistance until admission to a palliative care hospital for terminal thyroid cancer. Teriparatide therapy was continued for 18 months, followed by annual intravenous administration of zoledronic acid. No refracture was observed during the available follow-up period. The patient was followed for 5 years, during which no recurrence of infection or refracture was observed.

## Discussion

This case may represent a potential salvage approach for MRSA-infected distal radius non-union in a very elderly patient with severe osteoporosis. The principal clinical challenge was that infection eradication, stable fixation of small osteoporotic distal fragments, and preservation of wrist function all had to be achieved simultaneously. In this setting, standard revision internal fixation was considered less attractive because reimplantation of hardware into an infected osteoporotic metaphysis may increase the risks of recurrent infection and fixation failure. Bridging external fixation was also considered less desirable because it would likely have required prolonged wrist immobilization and might have compromised postoperative function. By contrast, a non-bridging circular construct offered multiplanar stability while permitting immediate motion of the wrist and forearm [1, 2].

The main value of this report lies in showing how staged wrist-preserving circular fixation can be combined with infection control and osteoporosis treatment in a single highly challenging case. However, the novelty should be interpreted carefully. Neither circular fixation, staged debridement, LIPUS, teriparatide, nor zoledronic acid is individually novel. Rather, this report describes the integration of these elements in one elderly patient with infected distal radius non-union and severe osteoporosis, a scenario that is rarely documented in the literature.

Another important point is the treatment rationale compared with alternative salvage options. Revision plate fixation was avoided because of the infected field, metaphyseal bone loss, and severe osteoporosis. Bridging external fixation would have controlled alignment but at the cost of wrist immobilization. Wrist arthrodesis might have provided durable stability and infection control, but it would have sacrificed residual wrist motion and was therefore less attractive in a patient whose functional independence was worth preserving. Resection arthroplasty was not favored because it would not have provided reliable structural restoration in this osteoporotic, infected, non-united distal radius. In our judgment, staged non-bridging circular fixation provided the best compromise between infection management, mechanical stability, and functional preservation in this patient.

The biological adjuncts also require cautious interpretation. In the present case, teriparatide was started preoperatively, LIPUS was used during the fixation period, and zoledronic acid was administered after completion of the anabolic phase. This sequence is biologically plausible, and it is consistent with the concept of stimulating bone formation first and then preserving skeletal gains [4, 5]. Nevertheless, the relative contribution of each intervention cannot be determined. The favorable outcome cannot be attributed specifically to teriparatide, LIPUS, zoledronic acid, or any putative synergy among them [6–10]. These adjuncts should therefore be described as potentially contributory rather than proven therapeutic drivers.

The functional outcome is another strength of this case, although it should also be presented with balance. The final DASH score of 10.7 was favorable, and the preserved forearm rotation shown at final follow-up supports the practical advantage of avoiding wrist-bridging fixation. At the same time, this is a single case, and the absence of serial structured patient-reported outcome collection or more comprehensive comparative data limits the strength of inference regarding functional superiority.

This report should be interpreted as hypothesis-generating rather than confirmatory. Its scientific value lies in demonstrating feasibility and providing a treatment concept for a rare and difficult clinical problem. Whether staged non-bridging circular fixation combined with systemic bone-health optimization offers advantages over more conventional staged revision methods will require larger case series, multicenter registries, or comparative observational studies.

## Conclusions

Staged non-bridging circular external fixation combined with sequential osteoporosis therapy may represent a potential salvage approach for MRSA-infected distal radius non-union in severely osteoporotic elderly patients, particularly when infection control, fixation stability, and preservation of wrist motion must all be considered. This approach enables stable fixation while allowing early mobilization and functional use of the wrist.

This case also highlights the importance of multidisciplinary management, including orthopedic surgery, infectious disease control, and osteoporosis treatment, in achieving successful outcomes in complex infected non-union cases.

However, as this is a single case treated with multiple simultaneous interventions, the findings should be interpreted cautiously and regarded as hypothesis-generating rather than evidence of efficacy of any specific component.

### Limitations

This report has several limitations. First, it describes a single case, and therefore its generalizability is inherently limited. A favorable outcome in one patient does not establish reproducibility across other patients with infected distal radius non-union and severe osteoporosis. Second, because multiple interventions were used in combination, including staged debridement, non-bridging circular external fixation, bone grafting, LIPUS, teriparatide, and subsequent zoledronic acid, the relative contribution of each component cannot be determined. Third, although the final DASH score was favorable and clinically meaningful function was preserved, more comprehensive longitudinal functional assessments were not systematically collected. Fourth, we did not perform a formal economic analysis, and the cost-effectiveness of this staged approach remains unknown. Finally, this report should be regarded as hypothesis-generating. Further multicenter case accumulation will be necessary to determine whether this approach offers meaningful advantages over conventional staged revision strategies.

## Data Availability

All data generated or analyzed during this study are available from the corresponding author upon reasonable request.

## Abbreviations

AO/OTA: Arbeitsgemeinschaft fur Osteosynthesefragen/Orthopaedic Trauma Association
CRP: C-reactive protein
CT: Computed tomography
DASH: Disabilities of the Arm, Shoulder, and Hand
LIPUS: Low-intensity pulsed ultrasound
MRSA: Methicillin-resistant Staphylococcus aureus
VAS: Visual analogue scale
WBC: White blood cell count
YAM: Young adult mean
